# Evaluating video and hybrid group consultations in general practice: mixed-methods, participatory study protocol (TOGETHER 2)

**DOI:** 10.3310/nihropenres.13584.1

**Published:** 2024-05-10

**Authors:** Chrysanthi Papoutsi, Gary Abel, Cynthia Iglesias, Jackie van Dael, Claire Reidy, Stuart D Faulkner, Helene Raynsford, Michele Siciliano, Luis Beltran Galindo, Vijay Gc, John Campbell, Trisha Greenhalgh, Sara E Shaw

**Affiliations:** 1 Nuffield Department of Primary Care Health Sciences, University of Oxford, Oxford, England, OX26GG, UK; 2 Department of Health and Community Science, Smeall Building, St Luke’s Campus, University of Exeter, Exeter, England, EX1 2LU, UK; 3 Department of Health Sciences, Seebohm Rowntree Building, University of York, York, England, YO10 5SQ, UK; 4 Independent public contributor, UK, UK; 5 School of Human & Health Sciences, University of Huddersfield, Huddersfield, England, UK

**Keywords:** group consulting, remote care, general practice, invisible work, lived experience

## Abstract

**Background:**

General practice is facing an unprecedented challenge in managing the consequences of the pandemic. In the midst of a policy drive to balance remote and in-person service provision, substantial workload pressures remain, together with increasing prevalence of long-term conditions, and declining staff numbers and morale. To address these challenges, some practices in the UK have been delivering video and hybrid group consultations (VHGCs). Despite positive initial findings and enthusiasm, there are still gaps in our understanding of the influence VHGCs have on patient experience, healthcare utilisation, quality, safety, equity and affordability.

**Objectives:**

To generate an in-depth understanding of VHGCs for chronic conditions in general practice, surface assumptions and sociotechnical dynamics, inform practice and extend theorisation.

**Methods:**

Mixed-methods, multi-site research study using co-design and participatory methods, from qualitative, quantitative and cost-related perspectives. WP1 includes a national, cross-sectional survey on VHGC provision across the UK. In WP2 we will engage patients and general practice staff in co-design workshops to develop VHGC models with emphasis on digital inclusion and equity. In WP3 we will carry out a mixed-methods process evaluation in up to 10 GP practices across England (5 sites already running VHGCs and 5 comparison sites). Qualitative methods will include interviews, focus groups and ethnographic observation to examine the experiences of patients, carers, clinical and non-clinical NHS staff, commissioners and policy-makers. Quantitative methods will examine the impact of VHGCs on healthcare utilisation in primary and secondary care, patient satisfaction, engagement and activation. We will also assess value for money of group and individual care models from a health economics perspective.

**Conclusions:**

We aim to develop transferable learning on sociotechnical change in healthcare delivery, using VHGCs as an exemplar of technology-supported innovation. Findings will also inform the design of a future study.

## Plain Language Summary

**Aim:**

To better understand how group consultations for chronic conditions can benefit patients and the health service when delivered via video and/or in-person in general practice.

**Background:**

Before the pandemic, group consultations were starting to gain ground in the UK as a new way of delivering clinical care to multiple patients at the same time, with potential benefits resulting from peer support and time efficiencies. When in-person care was restricted due to Covid-19, clinicians started delivering group consultations over video. Despite significant interest, we still know little about how group consultations delivered over video or hybrid models (combining video and in-person sessions) can be best implemented.

**Research methods:**

In up to 5 GP practices across England, where group consultations are already being delivered, we will evaluate how these new approaches to clinical care are implemented, and how they may support an inclusive service that engages patients with different needs and preferences. The evaluation will include interviews with patients, carers, NHS staff, policy-makers and commissioners, as well as group discussions and observations, including research led by patients themselves. We will also collect numerical data on the number and type of patients attending, whether they are more satisfied or confident with their self-management, or less likely to need to go to hospital. We will explore costs associated with these new ways of delivering care and will develop comparisons to face-to-face individual appointments. We will also work with comparable clinical sites only delivering one-to-one appointments, to collect numerical data on patient attendance, satisfaction and use of health services. With the involvement of our patient and public involvement (PPI) group, we will bring together our data to develop practical knowledge.

## Introduction

Efforts are constantly underway to reconfigure healthcare provision, more recently driven by immense demand pressures and the need to provide good patient care with limited resources ^1, 2^. New service models often rely on digital health innovations in the hope that technology-supported care will contribute to streamlined workloads, better prioritisation of patient need, and time savings, among other benefits ^3^. In the UK this became particularly pronounced in the context of Covid-19 when ‘remote-by-default’ care captured the imagination of policy-makers (until this policy was later reversed following public outcry) ^4– 6^. It is now becoming more widely understood that ‘remote services are difficult to set up, technically challenging, may increase workload at a stressful time, and could worsen health inequities’ ^7^.

Alongside remote care, the role of ‘lived experience’ has also received significant attention in recent decades. Patient and public involvement (PPI), peer group and ‘expert patient’ programmes all aim to harness patient ‘experiences’ as a resource towards healthcare delivery and improvement ^8, 9^. Although a welcome shift from dominant paternalistic models viewing patients as passive recipients of care, an increased focus on ‘lived experience’ also involves a number of assumptions about whose experiences count, how these can be articulated and in what ways they become meaningful (or not) in the context of healthcare delivery ^10– 12^. The ‘diversity and plurality of forms and articulations of knowledge that characterise experiential knowledge, as well as the gradual, dynamic and entangled process that leads from experience to knowledge and expertise’ are often neglected ^10^.

In Together 2 we are studying video and hybrid group consultations, a relatively novel way of care provision combining the logics outlined above: a) an attempt to harness the potential of remote care while b) foregrounding experiential knowledge and sharing as a resource for service provision. In the UK, video and hybrid group consultations started to gain traction during the Covid-19 pandemic, where physical distancing invited imaginative solutions to care delivery. These built on existing models and approaches to in-person group consulting pre pandemic where small groups of patients came together to have their clinical consultations at the same time (e.g. diabetes annual review in general practice), rather than in separate individual appointments. Combining clinical consultation with education and peer support, group consultations have been delivered in a variety of formats for patients with different long-term conditions or shared health concerns ^13, 14^.

A large part of the literature on group consultations has so far focused on assessing potential to improve clinical outcomes for specific conditions and to influence measures such as patient ‘satisfaction’ (e.g. see 15, 16). Although some studies provide positive findings, especially in clinical areas where patient self-care is important (e.g. diabetes), these tend to focus on face-to-face delivery, report on group consultations conducted as part of randomised controlled trials rather than standard clinical care, or have been carried out in contexts other than the UK ^15, 17– 21^. Only a small number of studies have focused (wholly or partly) on video group consultations in UK settings, including during the pandemic, and have provided insights in relation to facilitators/barriers or patient perceptions (e.g. see 16, 22, 23). Yet, more research is needed to understand whether and how group consultations, when provided remotely, can become embedded over time in different healthcare settings still recovering from the pandemic. Rich detail of the complexity of group consulting provision is only starting to emerge ^14, 24– 26^; there is little theorisation of the role patients themselves (and other critical actors) play in group sessions and the ways good group-based care relies on the capacity to manage invisible work and mobilise lived experience.

In Together 2 we seek to generate an in-depth understanding of different modes of remote group consulting (video and hybrid), surface assumptions and sociotechnical dynamics underpinning this relatively novel way of providing care, inform practice and extend theorisation. Informed by our previous work on in-person diabetes group consultations for socioeconomically disadvantaged young people in secondary care (NIHR HSDR-funded, 2016-21) ^25, 26^ and in-pandemic research on video group consultations in general practice (Health Foundation-funded, 2020-21) ^27^, the following research questions act as our starting point:

1. What is the feasibility and acceptability of video and hybrid group consultations to different population groups? What are their (perceived) implications for access, efficiency and safety in general practice?

2. How do patients, carers and NHS staff experience these new models of care, compared to standard one-to-one service provision and in-person group consultations?

3. What are the costs of introducing video and hybrid group consultations? What is the organisational impact on general practice and other system-level stakeholders?

4. What would be the optimal design of a large-scale evaluation to assess clinical benefits and key outcome/activity parameters?

## Protocol

### Patient and Public Involvement (PPI)

This study follows from previous research (as explained above) where PPI contributors had been involved and helped shape the research questions and design set out in this protocol. PPI contributors supporting this study include people with a range of conditions, caring responsibilities (including for family members who do not speak English), ages, genders, and ethnicities. Members of the group meet formally every ~6 months with individual conversations organised between meetings as needed to discuss specific topics in more detail or where PPI contributors find it easier to engage separately. The evaluation will be underpinned by participatory principles, including working with PPI contributors on, e.g. setting the agenda for co-design, reviewing recruitment materials, helping with data interpretation and developing patient-facing guidance documents.

### Study design

This mixed methods project includes three overlapping and interlinked work packages: A) Cross-sectional, UK-wide survey on group consulting provision over time (WP1), B) Co-design with patients and general practice staff with emphasis on inclusion and equity (WP2), C) Mixed methods, multi-site process evaluation in up to 5 case and 5 comparison sites (primarily GP practices in England) to generate actionable learning and in-depth understanding of implementation, from qualitative, quantitative and cost-related perspectives (WP3).

### A. National scoping survey (WP1)

We plan a national scoping survey of existing and emerging group consulting service models across care levels in the UK. The scoping survey will provide a picture of how group consulting was deployed as a remote model of care in the context of the pandemic (2020–21), and how it has been used in the recovery context (2021–22), including the types of conditions and patient populations for which it has been used, the types of consultations carried out in groups (e.g. annual reviews, health checks), frequency and format of sessions (e.g. in-person or remote delivery mode, inclusion of underserved communities), staff training, and key implementation opportunities and challenges, with a particular focus on inclusion and equity.

This online questionnaire will be developed using the JISC online surveys 2 platform ( Google Forms is a potential open-access alternative) at the University of Oxford and will extend a survey instrument used in a previous study on video consultations across the UK in 2020 ^28^. Data will be exported to Stata v17.0 for analysis ( R is a potential open-access alternative). Specifically in relation to GP practices (subject to sufficient number of responses), we will examine associations between the historical deployment of group consulting, and patient experience as measured by the national GP Patient Survey, focusing on key conditions for which practices report carrying out group consultations (such as asthma, diabetes and cancer) and restricting data to those patients who self-report one of these conditions. Further comparisons between practices carrying out group consultations and those not doing so will be made on the basis of practice characteristics such as practice size, workforce, deprivation, rurality and clinical quality as measured by the QOF. Analysis of these differences will be made using mixed effects linear logistic models as appropriate to the outcome. A random effect model will be used to assess differences between practices unexplained by VHGCs or chance and will provide a context for the size of differences associated with VHGCs. Free text comments will be analysed using a combination of qualitative and quantitative content analysis.

The survey will be distributed through multiple channels, including the Future NHS collaboration platform (e.g. community on remote and group consulting), NHS agencies and professional bodies (e.g. NHSE, NHS Scotland, NHS Wales, NHS Northern Ireland, Health Education England, RCGP, RCN), training programmes on group consulting, academic primary care networks and social media (e.g. Twitter). We will capitalise on existing professional networks with practitioners to ensure broad reach and expedite learning about existing and emerging group consulting models. 

### B. Iterative co-design (WP2)

The participatory co-design phase will focus on group consulting delivered remotely in general practice, i.e. video and hybrid formats (VHGCs). Drawing on principles of Experience-Based Co-Design (EBCD) ^29^ we will use excerpts from de-identified interview data with patients and staff and recordings of group consultations (see next section) to trigger conversation and exchange in co-design workshops. To ensure co-design covers all areas where implementation and sustained use may encounter challenges, we will draw on the 7 domains of the NASSS (non-adoption, abandonment, scale-up, spread, and sustainability) framework, an evidence-informed approach to guide thinking on the implementation and sustainability of healthcare innovations ^30^. This will help co-design participants focus discussions around opportunities for improvement and better integration of group consulting in the health service.

Co-design will involve up to a total of 40 patients, carers, clinical and non-clinical general practice staff in 4 virtual workshops. These will be supplemented with 10 individual patient interviews (in-person/phone) to delve into more detail on sensitive topics and engage vulnerable or digitally excluded groups (e.g. those with sight loss, hearing loss and learning difficulties). The co-design process will be iterative, focusing on how VHGCs can be delivered to meet patient and staff needs in efficient and inclusive ways. We will draw on creative and visual techniques (e.g. persona development, journey mapping, storyboards) to facilitate discussion and collaborative design (with support from Design Science, an external design agency). Outputs will include practical guidance on setting up and delivering VHGCs.

### C. Process evaluation (WP3)

The mixed methods process evaluation will use qualitative, quantitative and cost-related measures, based on a comparative case study design in up to 10 clinical sites in England (5 case and 5 comparison sites, primarily from general practice). Case sampling will focus on maximum variation in characteristics such as geographical location, size and digital maturity of the clinical site (digital readiness, capability and infrastructure), patient characteristics (ethnic background, digital literacy/confidence, deprivation) and VHGC delivery format and focus.

### Qualitative methods

***i. Patient participants***

In up to 5 case sites we will carry out semi-structured, qualitative interviews with 40–50 patients and carers (~10 per case site), seeking to understand their views and experiences (also using interpreters where needed, to account for ethnic diversity). We will engage patients and carers who have taken part in VHGCs, as well as those who declined participation or were unable to attend sessions (e.g. due to digital exclusion). A subset of participants will be invited to follow-up interview 12 months later to reflect on longitudinal changes. Interviews will follow a flexible topic guide, will last 45–60mins, and will take place remotely (on the phone or video) or at participants’ homes, their GP practice or another preferred location.

To allow participants to collectively debate their views on VHGCs, we will conduct 4–6 focus groups, each involving 6–8 patients and carers (n=24–48) who have not already participated in interviews. Each group will last 60–80 minutes and will be led by an experienced moderator using a flexible topic guide. We will aim to align some of these focus groups with existing VHGC sessions (especially when these are designed to be short) to capture immediate thoughts and feedback patients might have on what has gone well or less well.

We will recruit patients across sociodemographic and ethnic backgrounds, health needs and conditions, and with different levels of self-assessed digital literacy/confidence. Groups with higher general practice consultation rates will be particularly targeted (asking each practice which groups tend to make most use of services locally), including those with long-term conditions, those with multi-morbidities, and people from underinvested communities.

***ii. Clinical and non-clinical NHS staff***

Recruitment will primarily focus on case sites, where we expect to engage at least 4–6 staff per site in semi-structured interviews (n=20–30 in total) to better understand VHGC delivery in the contact of broader organisational routines. This will include health professionals (GPs, trainees, practice nurses, healthcare assistants, pharmacists etc), practice managers, administrators, reception and IT staff. A subset of staff participants will be interviewed twice over 12 months, to be able to follow up on growing familiarity with VHGCs and any longer-term impacts on the service. Interviews will last 45 mins and will be carried out either in-person or on the phone/video. An indicative topic guide will be followed, tailored to different interviewees.

We will also carry out ethnographic observation focusing on back-end operational and technical processes required to run VHGCs, to enrol patients in VHGCs and support their participation (at least 2 visits per case site over 12 months). The team will observe 25 VHGCs (virtually or in-person) across the 5 case sites (~5 each), using video or audio recordings and field notes. Any documentation, VHGC materials developed by the practice, significant event details or other relevant, de-identified records will be retained for analysis.

***iii. National and local policy-makers and commissioners***

To further our understanding of evolving policy and guidance, national-level incentives and regulation, we will conduct semi-structured, elite interviews with 5–10 key national decision-makers (e.g. NHSE). At a local level we will interview 5–7 commissioners across the 5 case site localities and observe relevant meetings, to gain further insights on VHGC impact on service planning and access. Interviews will last 45mins and will be carried out in-person or on phone/video. Relevant policy reports, public-facing content and other relevant materials will be retained for analysis.

With informed consent (written or verbal), qualitative interviews, group discussions and a sample of VHGCs will be audio or video recorded, and professionally transcribed. Field notes will be taken during observation, and photos will be used to illustrate back-end operational processes. All data will be de-identified and uploaded to NVivo 12 to support data management ( QualCoder is an open access alternative). Following data familiarisation and guided by our research questions, we will take an iterative approach to theory-informed, conceptual data coding in the first instance, following inductive, deductive and abductive approaches. We will compare and contrast data between cases, to develop an in-depth understanding of sociotechnical processes underpinning VHGC delivery and participation. Analysis will take place in parallel to data collection with a view to developing new theoretical insights. 

### Quantitative methods

***i. Description of VHGC delivery and resource use***

In each of the case sites we will collect descriptive data on the way VHGCs are delivered and the resources needed. These data will include the number and type of VHGC sessions, the number of patients participating in each VHGC and the number of patients invited to take part who choose not to participate. The data will be collected by the practice with support from the study team using structured proformas on a monthly basis. We will also collect key data, for those consultations observed by researchers, on timing and duration of VHGCs, resources needed (e.g. staff members) to deliver the sessions, and time and resources required to prepare for and follow up from each VHGC. The exact data required may depend on the way in which a practice chooses to deliver VHGCs but will likely include, the time at which the online session is started (e.g. by the ‘facilitator’), the time at which patients join the session, time spent facilitating technical issues, the time when the clinician joins the session (which in current VHGC models may be some time later than the patients), the time when the clinician leaves the session and the time when the session is ended. By examining this data we will gain an understanding of the time burden experienced by patients, GPs and other clinical and non-clinical staff, and how this changes over time.

***ii. Impact of VHGCs on patient experience, satisfaction and quality of life***

We aim to recruit up to 50 patients per practice who will consent to participate in a survey questionnaire comprising patient experience, satisfaction and healthcare-related outcome components, and to allow research access to their healthcare utilisation data. The survey will be administered online and offline, immediately following VHGC attendance (through a link shared on the same platform, e.g. Microsoft Teams, or in paper format for those attending in person), and will examine both patient experiences which relate directly to VHGCs (e.g. satisfaction with care received, inclusion and access implications, quality and safety of care, need for additional follow-up, travel time saved, time off work avoided etc.) as well as to wider experiences of healthcare in the practice (e.g. satisfaction with 1:1 care, perceptions on access, quality and safety). To develop the survey questionnaire, we will draw on questionnaires piloted in our previous research on patient experiences with video consultations and group clinics. We will also adopt or adapt items from existing surveys such as the GP Patient Survey ( https://gp-patient.co.uk/) and the Primary Care Outcomes Questionnaire (PCOQ) ^31^. We also expect new items to be developed. The findings will be used in descriptive analyses to provide a quantitative overview of patient experience of VHGCs.

In addition to the case sites, we will recruit an equal number of comparison practices where VHGCs are not being employed (matched with case sites on location, setting, size, deprivation, QOF). As with case sites, we will aim to recruit 50 patients from each comparison site with recruitment targeted at similar patients as those taking part in VHGCs in terms of age, gender and chronic conditions treated. They will receive a shorter version of the patient survey used in case sites with questions relating only to wider experiences of healthcare at their practice and will be asked to consent to research access to their healthcare utilisation data. Our team will also provide support to any comparison or case sites who choose to administer the Patient Activation Measure (PAM) in the context of local service improvement.

***iii. Impact of VHCGs on healthcare utilisation***

We will work with practices to collect data on patients’ primary care utilisation including the number, type (staff members involved, e.g. GP, nurse or other healthcare professional, and mode of consultation e.g., in-person, telephone, online) and duration of appointments. We will also obtain secondary care utilisation data from NHS Digital using Hospital Episode Statistics (HES) data. Comparisons will be made between patients at case and comparison sites in terms of number and duration of primary care consultations, number of A&E attendances, outpatient appointments (including numbers or proportion of eligible patients waiting more than six months), hospital admissions, unplanned hospital admissions and hospital admissions for ambulatory care sensitive conditions (conditions for which admissions could, in principle, be avoided by good primary care ^32^). Mixed effects models, with random intercepts for practice, will be used to analyse these data with the form of the model appropriate to the outcome (i.e. Poisson models for count of appointments/admissions and linear models for consultation durations). Adjustments will be made for patient factors including age, gender, deprivation, ethnicity and comorbidity status. Given the exploratory nature of this work we have not performed any formal power calculations and will interpret the results accordingly. Rather than focus on the presence or absence of statistically significant differences between groups, the analysis will instead examine confidence intervals as a guide to potential differences between the groups, to guide a larger, definitive study.

### Health economics evaluation

To assess the value for money of GP models of healthcare provision with and without VHGC we will conduct an early health economic evaluation (HEE) – in the form of a model based cost-effectiveness analysis – from the perspective of the NHS and Personal Social Services. The cost-effectiveness analysis will complement the qualitative and quantitative analyses conducted in WP1-3. The health economics (HE) team will contribute to the development of quantitative and qualitative data collection tools to be used in WP1-3. Findings from WP1 (scoping survey) and WP2 (co-design) will inform the process of: i) defining the decision problem for the early economic evaluation ii) conceptualising a decision model structure that represents the decision problem; and iii) generating a set of initial model inputs for a preliminary evaluation of the decision model. Furthermore, in preparation for a large definitive trial, the early HEE study will ascertain the feasibility of (and best practice for) collecting data – at GP practice level – on healthcare resource use (e.g. venue, staffing, consultations’ duration, set-up time, technology costs) and patient reported outcomes (e.g., health related quality of life, satisfaction) associated with the delivery of VHGCs.

Decision Model: conceptualisation and development of the decision model ^33^ will be guided by the NASSS domains (i.e. clinical condition, technology, value proposition, adopter system, organisation, wider system and temporal change ^34^ and their recent adaptation for remote consultations ^35^). The process of conceptualising and developing the decision model will involve decomposing and characterising discrete elements of GP models of care. The decision model will enable estimation of the value for money of a GP model of healthcare provision that includes alternative types (i.e. one-to-one, group) and modes (i.e. one-to-one, group, in-person, home, video or hybrid) of GP consultations including VHGC, compared to a GP model of healthcare provision that does not include VHGC.

Model inputs: the focus of the VHGC (e.g., diabetes annual reviews; peri and post menopause consultations) will be defined in consultation with case and comparison sites in WP3. A micro-costing analysis of VHGCs will be conducted in one of the five case sites in WP3 GP. Data on consultations (e.g. number and type of VHGCs) will be collected prospectively. The main source to estimate the unit cost of different types and modes of GP consultations will be the Unit Costs of Health and Social Care Manual ( https://www.pssru.ac.uk/unitcostsreport/). Over an up to 6-month period, all patients attending a GP consultation (any type and mode, but with the same focus as VHGCs) in one case and one comparison site will be invited to complete an online Measures of Health Benefit survey. This will include questions on utility-based health-related quality of life (i.e. EQ-5D-5L) ^36^, patient satisfaction (i.e. SOPS adaptation) ^37^ and personal details (e.g. age, weight, height).

Initial model inputs will be based on estimates of consultation costs, utility, and satisfaction. Given the limited data available to populate our decision model, and the need to make a number of expert-informed structural assumptions, we expect the results and conclusions derived from the early HEE to be subject to uncertainty. The impact of this on the decision uncertainty to consider VHGC a cost-effective addition to exiting models of GP healthcare provision will be explored using value of information analysis (VOI) ^38^. This methodology will enable us to identify the model parameters (and structural assumptions) considered to be the key drivers of decision uncertainty. Results from the VOI analysis will be used to inform the design of a future definitive study of a GP model of healthcare provision with VHGCs.

### Data synthesis and engagement with theory

We are using the Non-adoption, Abandonment and Challenges to Spread, Scale-Up and Sustainability (NASSS) framework to inform study design and data collection on the multiple interacting influences affecting implementation and sustainment of VHGCs ^34^. The framework will also contribute in crafting narrative case studies combining qualitative, quantitative and cost-related data for each site where VHGCs have been taking place. Integration of qualitative and quantitative data will inform the development of a theory of change, i.e. set of assumptions on successful delivery of VHGCs and un/anticipated implications in practice. Extending previous research on in-person and video group consulting ^26, 27^, we will draw on a number of theoretical threads from science and technology studies, medical sociology and organisational theory to underpin data analysis and synthesis. This includes theory on: a) burden of treatment and patient ‘work’ ^39^, b) digital exclusion and intersectionality ^38, 40^, c) health service complexity ^41, 42^ and d) experiential knowledge ^10, 12^.

## Ethics and governance

Approval has been granted by the London Hampstead Research Ethics Committee and UK Health Research Authority, reference number 22/PR/0277 (19 May 2022), and subsequent amendments. The study is overseen by an independent external advisory group with a lay chair and diverse representation from policy, clinical care, the commercial sector, and people with experience on patient advocacy groups or regulatory bodies.

## Outputs and dissemination

We plan a number of key outputs including: a) empirical academic articles and conference presentations, b) co-designed guidance for patients and service providers on developing effective and inclusive video and hybrid group consulting, c) policy briefings around supporting equitable group-based digital services. We will facilitate knowledge sharing and capacity building across participating sites through joint co-design sessions, learning reports, and virtual learning events to share formative and summative evaluation findings and drive local improvement.

## Conclusions

We aim to develop transferable learning on sociotechnical change in healthcare, using VHGCs as an exemplar of technology-supported innovation. Findings will inform policy and practice, as well as the design of a future definitive study.

## Data Availability

No data are associated with this article.
