# Supplementary material for: Evaluating video and hybrid group consultations in general practice: mixed-methods, participatory study protocol (TOGETHER 2)
Source: NIHR Open Res. 2024 Sep 18;4:30. Originally published 2024 May 10. [Version 2] doi: 10.3310/nihropenres.13584.2 (PMC11415752; doi:10.3310/nihropenres.13584.2)
Supplement: Peer review history

# Reviewer report: Reviewer response for version 2

**DOI:** 10.3310/nihropenres.14918.r32965

**Published:** 2024-09-20

**Authors:** Jennifer Job

**Affiliations:** 1 Mater Research Institute-University of Queensland, Herston, Australia

Thank you. I approve the manuscript for indexing.

 Kind regards,

 Jenny

Is the study design appropriate for the research question?

Yes

Is the rationale for, and objectives of, the study clearly described?

Yes

Are sufficient details of the methods provided to allow replication by others?

Partly

Are the datasets clearly presented in a useable and accessible format?

Not applicable

Reviewer Expertise:

Primary care research, implementation science

I confirm that I have read this submission and believe that I have an appropriate level of expertise to confirm that it is of an acceptable scientific standard.

# Reviewer report: Reviewer response for version 2

**DOI:** 10.3310/nihropenres.14918.r32964

**Published:** 2024-09-19

**Authors:** Rasmus Dahl Larsen

**Affiliations:** 1 Southern Denmark University, Odense, Denmark

Approved

Is the study design appropriate for the research question?

Yes

Is the rationale for, and objectives of, the study clearly described?

Yes

Are sufficient details of the methods provided to allow replication by others?

Yes

Are the datasets clearly presented in a useable and accessible format?

Yes

Reviewer Expertise:

Specialist in General Practice, MD, Phd-student in Video group consultation in GP and partycipatory design.

I confirm that I have read this submission and believe that I have an appropriate level of expertise to confirm that it is of an acceptable scientific standard.

# Reviewer report: Reviewer response for version 1

**DOI:** 10.3310/nihropenres.14745.r31861

**Published:** 2024-06-13

**Authors:** Rasmus Dahl Larsen

**Affiliations:** 1 Southern Denmark University, Odense, Denmark

Thank you for the opportunity to review the study titled: Evaluating Video and Hybrid Group Consultations in General Practice: Mixed-Methods, Participatory Study Protocol. Overall, this PPI co-designed protocol is well-developed and appears very comprehensive, uncovering patients`, general practitioners', practice staffs and stakeholders’ thoughts and experiences with the use of video and hybrid group consultations in general practice and for which conditions group consultation has been used. Additionally, it addresses issues related to time resources and the economic aspects of using video and hybrid group consultations in general practice.

 The study is divided into three work packages, which is a logical approach. WP1 is exploratory, WP2 involves designing and testing, and WP3 focuses on further testing and evaluation. However, the connections (the common thread) between these packages could be made more visible, specifically if the work packages are interdependent and if yes, in what way.

 WP1:

 The questionnaire appears relevant for addressing clear questions regarding which chronic conditions and the overall setup of how video/hybrid consultations have been used and in which context. It also allows for comparisons with previous satisfaction surveys.

 Perhaps the authors could consider the following:

 - It might be interesting to involve PPI in the development and testing of this questionnaire.

 - Explain the purpose of the questionnaire and the specific information being gathered.

 - Describe how the data gathered in WP1 will be utilized and integrated into WP2.

 - Briefly explain what the GP survey is - As well as the OQF.

 - Consider using semi-structured interviews to further explore stakeholders’ answers from the questionnaire - what works and what doesn't, such as disease groups, practice and patient characteristics, etc. That could be a good preparation for WP2.

 WP2: Is very interesting and will reveal valuable insights about what works well in group settings for both patients and healthcare personnel. It is also beneficial to explore this for the vulnerable patient group, who can greatly benefit from peer-to-peer support. Perhaps the authors could consider the following:

- Provide a more detailed description of the workshop setup. Will it involve only patients?  discussing with other patients, or will there be mixed groups of patients and GPs, staff members and stakeholders?
- Is there a plan to conduct a mock-up remote group consultation and evaluation of it?
- Once again, how will the results from WP1 be integrated into WP2.
- How will the results from WP2 be used in WP3 – if at all?

WP3: The evaluation of the project appears interesting, with important evaluation points such as health economics, sociotechnical change and dynamics, and an analysis of remote group consultations.

 - Is it the aim to develop a "golden standard of remote group consultations" in WP2, and then test it and compare it with the five control GPs in WP3? Its not clear for the reader.

 - is it possible to estimate the expected data volume for the quantitative part? Can robust and meaningful conclusions be made based on the quantitative data set?

 - The above reflections are also relevant for ensuring an accurate health economic analysis.

 - It could be elaborated upon what is alluded at with “surface assumptions”. Moreover, “sociotechnical change” as a concept and the various theories mentioned at the end of the review could be described in more detail, not least why these are found relevant.  

 Overall:

 Furthermore, the authors could consider incorporating even more Patient and Public Involvement (PPI). For example, involving patients as co-creators in the development of the questionnaire in WP1, interview guide, and workshop, as well as in deciding which conditions and setups should be investigated, could provide valuable insights, and improve the study's relevance and impact.

Is the study design appropriate for the research question?

Yes

Is the rationale for, and objectives of, the study clearly described?

Yes

Are sufficient details of the methods provided to allow replication by others?

Yes

Are the datasets clearly presented in a useable and accessible format?

Yes

Reviewer Expertise:

Specialist in General Practice, MD, Phd-student in Video group consultation in GP and partycipatory design.

I confirm that I have read this submission and believe that I have an appropriate level of expertise to confirm that it is of an acceptable scientific standard.

# Response

**Published:** 2024-09-04

**Authors:** Chrysanthi Papoutsi

**Affiliations:** University of Oxford, Oxford, England, UK

Please see below our point-by-point response to reviewer 2 comments:

*Thank you for the opportunity to review the study titled: Evaluating Video and Hybrid Group Consultations in General Practice: Mixed-Methods, Participatory Study Protocol. Overall, this PPI co-designed protocol is well-developed and appears very comprehensive, uncovering patients`, general practitioners', practice staffs and stakeholders’ thoughts and experiences with the use of video and hybrid group consultations in general practice and for which conditions group consultation has been used. Additionally, it addresses issues related to time resources and the economic aspects of using video and hybrid group consultations in general practice.*

 Thank you

*The study is divided into three work packages, which is a logical approach. WP1 is exploratory, WP2 involves designing and testing, and WP3 focuses on further testing and evaluation. However, the connections (the common thread) between these packages could be made more visible, specifically if the work packages are interdependent and if yes, in what way.*

 Response: We have added further clarification: a) for WP1 ‘Survey results will inform subsequent phases of the study e.g. in terms of site selection’ in WP3, b) for WP2 ‘Co-design will iteratively inform the focus of data collection in WP3, especially qualitative and inclusion aspects, and emerging findings from WP3 will feed into the co-design workshops’, c) for WP3c the protocol already stated ‘Findings from WP1 (scoping survey) and WP2 (co-design) will inform the process of: i) defining the decision problem for the early economic evaluation ii) conceptualising a decision model structure that represents the decision problem; and iii) generating a set of initial model inputs for a preliminary evaluation of the decision model.’ Further details on interdependencies that are emerging as the project evolves will be included in the final findings paper.

*WP1:*

*The questionnaire appears relevant for addressing clear questions regarding which chronic conditions and the overall setup of how video/hybrid consultations have been used and in which context. It also allows for comparisons with previous satisfaction surveys.*

*Perhaps the authors could consider the following:*

*- It might be interesting to involve PPI in the development and testing of this questionnaire.*

 Response: As we explain the questionnaire extends a survey used in a previous study on video consultations in 2020 – both the previous study as well as the one described in this protocol have been informed by PPI contribution and this has included input to some of the survey questions, e.g. on inclusion. This is described in the findings paper for the study currently in development.

*- Explain the purpose of the questionnaire and the specific information being gathered.*

 Response: The protocol explains as follows: ‘The scoping survey will provide a picture of how group consulting was deployed as a remote model of care in the context of the pandemic (2020-21), and how it has been used in the recovery context (2021-22), including the types of conditions and patient populations for which it has been used, the types of consultations carried out in groups (e.g. annual reviews, health checks), frequency and format of sessions (e.g. in-person or remote delivery mode, inclusion of underserved communities), staff training, and key implementation opportunities and challenges, with a particular focus on inclusion and equity.’

*- Describe how the data gathered in WP1 will be utilized and integrated into WP2.*

 Response: We have now explained: ‘Survey results will inform subsequent phases of the study e.g. in terms of site selection’

*- Briefly explain what the GP survey is - As well as the OQF.*

 Response: We have provided relevant reference.

*- Consider using semi-structured interviews to further explore stakeholders’ answers from the questionnaire - what works and what doesn't, such as disease groups, practice and patient characteristics, etc. That could be a good preparation for WP2.*

 Response: The study includes relevant interviews in WP3.

*WP2: Is very interesting and will reveal valuable insights about what works well in group settings for both patients and healthcare personnel. It is also beneficial to explore this for the vulnerable patient group, who can greatly benefit from peer-to-peer support. Perhaps the authors could consider the following:*

*- Provide a more detailed description of the workshop setup. Will it involve only patients?  discussing with other patients, or will there be mixed groups of patients and GPs, staff members and stakeholders?*

 Response: We have added this is 2 workshops for patients and 2 for staff.

*- Is there a plan to conduct a mock-up remote group consultation and evaluation of it?*

 Response: This is an interesting idea – we used de-identified clips from WP3 to trigger discussion and will provide further explanation on this in the findings papers out of the study. The protocol states: ‘…we will use excerpts from de-identified interview data with patients and staff and recordings of group consultations (see next section) to trigger conversation and exchange in co-design workshops’

*- Once again, how will the results from WP1 be integrated into WP2.*

 Response: The results from WP1 mainly informed site selection and provided links to relevant stakeholders who were invited to the co-design workshops, as per the response in the relevant comment above.

*- How will the results from WP2 be used in WP3 – if at all?*

 Response: We have provided relevant clarification (as explained above): ‘Co-design will iteratively inform the focus of data collection in WP3, especially qualitative and inclusion aspects, and emerging findings from WP3 will feed into the co-design workshops.’

*WP3: The evaluation of the project appears interesting, with important evaluation points such as health economics, sociotechnical change and dynamics, and an analysis of remote group consultations.*

*- Is it the aim to develop a "golden standard of remote group consultations" in WP2, and then test it and compare it with the five control GPs in WP3? Its not clear for the reader.*

 Response: As the proposal suggests WP2 will develop practical guidance on setting up and delivering VHGCs – we do not believe there is a gold standard and only intend to develop guidance that staff and patients can use flexibly (and adapt) depending on their needs. We have now clarified this in the relevant section.

*- is it possible to estimate the expected data volume for the quantitative part? Can robust and meaningful conclusions be made based on the quantitative data set?*

 Response: We have provided indicative numbers based on experience – the study is not looking to draw definitive conclusions based on quantitative data, rather we are looking to consolidate data across methods supported by theory to enhance the value of this work. Group consultations remain a relatively new model of care with remote delivery reaching relatively low volumes, therefore we are not expecting to be able to reach significant numbers at this stage and the study has been designed accordingly.

*- The above reflections are also relevant for ensuring an accurate health economic analysis.*

 Please see response above.

*- It could be elaborated upon what is alluded at with “surface assumptions”. Moreover, “sociotechnical change” as a concept and the various theories mentioned at the end of the review could be described in more detail, not least why these are found relevant.*

 Response: We have clarified what we mean by sociotechnical change also in response to reviewer 1 comments.

 We have also elaborated that assumptions relate to what these models are meant to achieve and provided details on the theories mentioned: ‘This includes theory on: a) burden of treatment and patient ‘work’ related to the responsibilities placed on patients in the context of group consultations (38), b) digital exclusion and intersectionality around multiple dimensions that contribute to some people being served less well by remote means of group-based care  (39, 40), c) health service complexity including a departure from linear cause-effect relationships to recognising interdependence and emergence in group-based care delivery (41, 42) and d) experiential knowledge mobilised to support clinical care (10, 12)’.

*Overall:*

*Furthermore, the authors could consider incorporating even more Patient and Public Involvement (PPI). For example, involving patients as co-creators in the development of the questionnaire in WP1, interview guide, and workshop, as well as in deciding which conditions and setups should be investigated, could provide valuable insights, and improve the study's relevance and impact.*

 Response: Thank you for this helpful point – our evaluation is underpinned by participatory principles. In the protocol we have only provided some examples for PPI contribution. The findings papers will provide more detail on specific PPI activities, including the ones mentioned in the comment above.

# Reviewer report: Reviewer response for version 1

**DOI:** 10.3310/nihropenres.14745.r31862

**Published:** 2024-05-27

**Authors:** Jennifer Job

**Affiliations:** 1 Mater Research Institute-University of Queensland, Herston, Australia

Thank you for the opportunity to review this well written manuscript outlining a protocol for the evaluation of the benefits to patients of group consultations when delivered via video and/or in-person in general practice for multiple patients, with the same chronic conditions.

 This manuscript proposes a valuable body of work which will provide important data on patient experience, utilisation, safety, equity, and affordability of this model. The rationale and research questions are clearly defined.

 The definition provided in the plain language summary was useful for the reader, who may be unfamiliar with the model, to clarify the authors meaning of VHGCs. Would including this in the abstract highlight the focus for potential readers, particularly those unfamiliar with the model?

 Are VHGCs delivered by a one practitioner? GPs? Other health professionals?

 The term “sociotechnical change” was unfamiliar – I wonder if it would be useful to expand on this.

 Page 5:

 National GP patient survey – could you provide the reference for this when it is first mentioned on page 5.

 Could you provide a reference for QOF?

 Page 7:

 It would be interesting to know the proposed data collection methods e.g. spreadsheets, or an online portal?

 How will ‘deprivation’ be measured?

Is the study design appropriate for the research question?

Yes

Is the rationale for, and objectives of, the study clearly described?

Yes

Are sufficient details of the methods provided to allow replication by others?

Partly

Are the datasets clearly presented in a useable and accessible format?

Not applicable

Reviewer Expertise:

Primary care research, implementation science

I confirm that I have read this submission and believe that I have an appropriate level of expertise to confirm that it is of an acceptable scientific standard, however I have significant reservations, as outlined above.

# Response

**Published:** 2024-09-04

**Authors:** Chrysanthi Papoutsi

**Affiliations:** University of Oxford, Oxford, England, UK

Please see below our response to Reviewer 1 comments -

***Reviewer Comments: **Thank you for the opportunity to review this well written manuscript outlining a protocol for the evaluation of the benefits to patients of group consultations when delivered via video and/or in-person in general practice for multiple patients, with the same chronic conditions.* *This manuscript proposes a valuable body of work which will provide important data on patient experience, utilisation, safety, equity, and affordability of this model. The rationale and research questions are clearly defined.*

**Author Response: **Thank you

***Reviewer Comments: **The definition provided in the plain language summary was useful for the reader, who may be unfamiliar with the model, to clarify the authors meaning of VHGCs. Would including this in the abstract highlight the focus for potential readers, particularly those unfamiliar with the model?*

**Author Response: **Helpful suggestion – we have now included the definition in the abstract

***Reviewer Comments: **Are VHGCs delivered by a one practitioner? GPs? Other health professionals?*

**Author Response: **We have clarified in the introduction section: ‘Combining clinical consultation with education and peer support, group consultations are led by different health professionals (GPs, pharmacists, nurses) and have been delivered in a variety of formats for patients with different long-term conditions or shared health concerns (13, 14).’

***Reviewer Comments: **The term “sociotechnical change” was unfamiliar – I wonder if it would be useful to expand on this.*

**Author Response: **We have provided additional clarification: ‘In Together 2 we seek to generate an in-depth understanding of different modes of remote group consulting (video and hybrid), surface assumptions and sociotechnical dynamics (the interplay between technical and social elements) underpinning this relatively novel way of providing care, inform practice and extend theorisation.’

***Reviewer Comments: **Page 5: National GP patient survey – could you provide the reference for this when it is first mentioned on page 5.*

**Author Response: **We have provided relevant reference.

***Reviewer Comments: **Could you provide a reference for QOF?*

**Author Response: **We have provided relevant reference.

***Reviewer Comments: **Page 7: It would be interesting to know the proposed data collection methods e.g. spreadsheets, or an online portal?*

**Author Response: **It is not clear to us what part of page 7 this comment refers to but we have added clarification (where it seemed relevant) that we are using Word proformas.

***Reviewer Comments: **How will ‘deprivation’ be measured?*

**Author Response: **We have clarified that we are using the index of multiple deprivation and provided reference: https://data.cdrc.ac.uk/dataset/index-multiple-deprivation-imd
